# Go, Flight

**DOI:** 10.1172/jci.insight.185748

**Published:** 2024-09-10

**Authors:** Oliver Eickelberg

This issue of *JCI Insight* marks the transition of the editorial board from the University of Michigan to the University of Pittsburgh. The University of Michigan team, led by Kathleen L. Collins, MD, PhD, have masterfully extended the reach and scope of the Journal during their tenure, which followed *JCI Insight’s* founding on January 21, 2016, under the leadership of Howard A. Rockman, MD, of Duke University. In the inaugural issue’s editorial (1), Executive Editor Sarah Jackson highlighted that by “creating this journal, we sought to provide an expanded forum for a wide range of preclinical, translational, and clinical research that uncovers new insights into the basis of disease and therapeutic approaches.” This mission still holds true today. The University of Pittsburgh team is honored to follow in the footsteps of the Duke University and University of Michigan teams to build the strong reputation of the highest quality science in disease-relevant fields of today’s medicine

*JCI Insight* is currently in its ninth year; hence it is, relatively speaking, in its childhood. Childhood is defined in fairly broad terms as “the time for cognitive, social, and emotional development”; hence efforts should be made for safe, loving, and secure environments (from the United Nations Convention on the Rights of the Child) (2). Under my leadership as editor in chief, the editorial team at the University of Pittsburgh will strive to provide this safe and secure environment for our authors and community of readers and constituents. With many scientific journals demanding ever-increasing workloads on authors (3), our team intends to make the publishing process easier, speedier, and less labor-intensive for the authors. We also have several new initiatives for our community of authors and readers. Let me introduce some of those we have launched and will continue to launch over the coming five years.

*We are pleased to announce that JCI Insight will now publish Research Letters*. These short reports feature content with a high degree of novelty and impact with disease-relevant findings that may not have fully explored mechanistic insights expected from a full-length article. We trust that this new category will particularly appeal to our early-career faculty, such as K awardees or equivalents, as well as American Society for Clinical Investigation (ASCI) Emerging-Generation (E-Gen) Award and Young Physician-Scientist Award (YPSA) awardees, who may not yet be able to tap into the full experimental armamentarium of a multi-award principal investigator’s lab but have important findings that will benefit the scientific community by publication.

*We will feature themed issues that highlight similar biological mechanisms across multiple organ systems*. We recognize the increasing importance of foundational biological processes that underlie multiple disease mechanisms. Stay tuned for themed issues on topic, including “Regenerative approaches to disease,” “Mechanisms of fibrosis across tissues,” “Impact of senescence on disease onset and outcome,” “Mechanisms of aging across organs,” and “Bioengineering approaches for regenerative medicine,” among other topics.

*We will facilitate effective and timely reviewing of submissions to JCI Insight by assigning a primary (handling) editor and a secondary (reviewing) editor*. Teamwork between two editors for each submission will facilitate reviewer identification and support decision-making in case reviewers disagree on the impact of a given submission. Such teamwork will also serve to reduce potential bias during the review process and enhance interaction between board members.

*We are pleased to support and promote early-stage investigators by including YPSA/E-Gen awardees as associate editors.* Such integration into the editorial team of *JCI Insight* will grow YPSA/E-Gen editors’ skill set, academic profile, and integration into the ASCI. Each YPSA/E-Gen editor will be mentored by the Chief or a Deputy Editor. Select members of the editorial leadership team (Chief, Deputy, and Executive Editor) will hold monthly meetings with the YPSA/E-Gen editors to facilitate their onboarding, enhance the decision-making experience, and provide essential input for challenges and opportunities during the editorial process.

*We will also now allow authors to upload reviews from other journals with their submissions*. The goal of this program is to streamline the review process, particularly when authors have completed revisions in response to prior peer reviews. The Editors will consider this information along with the manuscript in determining priority fit for *JCI Insight*. We may still need input from additional reviewers, but we hope that access to the prior reviews will expedite the evaluation of the work. 

*We will enhance the societal impact of JCI Insight by improving our presence on social media*. We hope to feature authors of select articles and tell the stories behind the published articles, including specific challenges and achievements. Look for us on X, Facebook, and Instagram, and tag us in your conversations about your work.

*In partnership with the JCI, we plan to implement a dual-journal submission option*. Papers designated for the dual-journal submission track will automatically be considered by *JCI Insight* in the event that the *JCI* rejects the manuscript. This process will save our community of authors and reviewers valuable time and help authors more quickly identify the journal that is the best fit for their work. Be on the lookout for more details about the dual-journal submission process soon.

These efforts will require a team of experts, which we are fortunate to have at the University of Pittsburgh. Our editorial team includes Deputy and Associate Editors, as well as Strategic Advisors with outstanding expertise and editorial experience that span the spectrum of basic, translational, and clinical research, including rapidly evolving disciplines such as bioengineering, systems biology/immunology, senescence, aging, and regenerative medicine. All of us share a passion for *JCI Insight* as a vessel to communicate the most important science of and to our ASCI members and the broader biomedical research community. I am fortunate to lead these efforts with the invaluable support of our Deputy Editors Stephen Chan, MD, PhD; Toren Finkel, MD, PhD; Anne Marie Lennon, MD, PhD; and Alison Morris, MD, MS. We are supported by a team of Associate Editors and Strategic Advisors, who will help implement the mission of *JCI Insight* by covering all disciplines of interest and expediting the review of submitted manuscripts. Most importantly, the journal is managed by our invaluable Executive and Senior Science Editors, Sarah Jackson and Corinne Williams, respectively. Together, we seek to achieve and maintain an average submission to decision deadline of 21 days or fewer and maintain a publication lag time (from acceptance to publication) of less than 2 months.

Importantly, *JCI Insight* will maintain strong connections with the *JCI*, ASCI’s flagship journal. We continue to have a strong interest in transferring manuscripts from the *JCI* for publication in *JCI Insight*. The editorial leadership team will assess the reviews (if available) from the *JCI*, invite papers of high interest, and expedite reviewing of those submissions based on existing reviews.

In conclusion, the two previous editorial teams have successfully launched *JCI Insight* to orbit. It is our aim to fuel its trajectory to even deeper reaches, and we will offer all of our expertise to do so. In a time when mechanistic investigations and publications face unprecedented challenges because of the advent of hypothesis-free approaches, the rise of predatory journals, and unreasonable burdens of publishing to authors, we strive to provide a platform supporting our biomedical researchers and physician-scientist community, publish investigations based on the scientific method, and feature stories of hope in times of funding challenges. Go, flight!

## Version 1 09/10/2024

Electronic publication
